# Specific vagus nerve stimulation parameters alter serum cytokine levels in the absence of inflammation

**DOI:** 10.1186/s42234-020-00042-8

**Published:** 2020-04-10

**Authors:** Téa Tsaava, Timir Datta-Chaudhuri, Meghan E. Addorisio, Emily Battinelli Masi, Harold A. Silverman, Justin E. Newman, Gavin H. Imperato, Chad Bouton, Kevin J. Tracey, Sangeeta S. Chavan, Eric H. Chang

**Affiliations:** 1 Laboratory of Biomedical Science, Feinstein Institutes for Medical Research, Northwell Health, 350 Community Drive, Manhasset, NY 11030 USA; 2 Institute of Bioelectronic Medicine, Feinstein Institutes for Medical Research, Northwell Health, 350 Community Drive, Manhasset, NY 11030 USA; 3 Donald and Barbara Zucker School of Medicine at Hofstra/Northwell, 500 Hofstra University, Hempstead, New York 11030 USA; 4 The Elmezzi Graduate School of Molecular Medicine, 350 Community Drive, Manhasset, NY 11030 USA

**Keywords:** Inflammatory reflex, Neuromodulation, Tumor necrosis factor, Interleukin-10

## Abstract

**Background:**

Electrical stimulation of peripheral nerves is a widely used technique to treat a variety of conditions including chronic pain, motor impairment, headaches, and epilepsy. Nerve stimulation to achieve efficacious symptomatic relief depends on the proper selection of electrical stimulation parameters to recruit the appropriate fibers within a nerve. Recently, electrical stimulation of the vagus nerve has shown promise for controlling inflammation and clinical trials have demonstrated efficacy for the treatment of inflammatory disorders. This application of vagus nerve stimulation activates the inflammatory reflex, reducing levels of inflammatory cytokines during inflammation.

**Methods:**

Here, we wanted to test whether altering the parameters of electrical vagus nerve stimulation would change circulating cytokine levels of normal healthy animals in the absence of increased inflammation. To examine this, we systematically tested a set of electrical stimulation parameters and measured serum cytokine levels in healthy mice.

**Results:**

Surprisingly, we found that specific combinations of pulse width, pulse amplitude, and frequency produced significant increases of the pro-inflammatory cytokine tumor necrosis factor (TNF), while other parameters selectively lowered serum TNF levels, as compared to sham-stimulated mice. In addition, serum levels of the anti-inflammatory cytokine interleukin-10 (IL-10) were significantly increased by select parameters of electrical stimulation but remained unchanged with others.

**Conclusions:**

These results indicate that electrical stimulation parameter selection is critically important for the modulation of cytokines via the cervical vagus nerve and that specific cytokines can be increased by electrical stimulation in the absence of inflammation. As the next generation of bioelectronic therapies and devices are developed to capitalize on the neural regulation of inflammation, the selection of nerve stimulation parameters will be a critically important variable for achieving cytokine-specific changes.

## Background

Electrical stimulation is an important neuromodulation technique used to activate nerves and muscles within the body. When electrical current is applied to nerve tissue using electrodes, there are multiple variables that determine whether the desired neural fibers are activated. In general, fiber activation by electrical stimulation follows a recruitment order from largest to smallest, with the smallest diameter fibers requiring the highest stimulation current levels (Baratta et al., 1989; Blair et al., 1933; Fang and Mortimer, 1991). Because diverse fiber types in the peripheral nervous system innervate different target organs and exhibit distinct functions, this fiber recruitment principle is important to achieve the desired physiological changes from activating nerves (Gorman and Mortimer, 1983). Electrical stimulation parameters such as output frequency, current, duration, and amplitude, are important determinants for achieving selective and efficient nerve activation (Grill, 2015).

In the vagus nerve, there are three main fiber types: A-, B-, and C-fibers that can be distinguished on the basis of axon diameters, myelination, conduction velocity and stimulation thresholds for activation (Heck et al., 2002; Groves and Brown, 2005). This mixed nerve carries sensory afferent and motor efferent signals between the brain and the body to mediate vital functions of the autonomic nervous system (Bonaz et al., 2013; Tracey, 2002). A large body of preclinical studies and emerging clinical evidence indicates that electrical stimulation at the cervical vagus nerve is able to change the body’s immune response to injury or infection, specifically by reducing the levels of certain serum cytokines that are important mediators of inflammation in the body (Andersson and Tracey, 2012; Borovikova et al., 2000). This stimulation of the vagus nerve regulates cytokine release from the spleen through activation of the *inflammatory reflex*, thereby protecting against lethality in models of systemic inflammation (Borovikova et al., 2000; Chavan et al., 2017; Tracey, 2002). Early clinical evidence for the therapeutic efficacy of this electrical vagus nerve stimulation has shown promise to treat patients with chronic inflammatory disorders (Bonaz et al., 2016; Koopman et al., 2016). Previous studies have also demonstrated that electrical stimulation parameters can be modified to intentionally elicit different physiological effects, such as the separation of anti-inflammatory and cardioinhibitory effects (Huston et al., 2007). The anti-inflammatory effects of vagus nerve stimulation have been attributed to A- and B-fiber activation, while the cardioinhibitory effects are thought to be mediated by only B-fibers (Huston et al., 2007; Olofsson et al., 2015; Yoo et al., 2016).

As nerve stimulation parameters affect differential fiber recruitment, we reasoned that specific stimulation parameters might affect serum cytokine levels in a parameter-dependent fashion. We modified the output of our electrical stimulator to select frequencies, amplitudes, and pulse widths predicted to differentially activate different classes of fibers within the vagus nerve. Here we observed that specific combinations of frequency, pulse width, and pulse amplitudes either increase or decrease certain cytokines, such as tumor necrosis factor alpha (TNFα) and interleukin 10 (IL-10) in healthy mice.

## Methods

### Animals

Naïve male BALB/c mice (8 to 12 weeks old) were obtained from Charles River Laboratories (Wilmington, MA, USA) and acclimated for at least 1 week before conducting experiments. Animals were housed on a 12:12 h reverse light/dark cycle at 23^o^ C and relative humidity 30–70%. Mice were housed with ad libitum water and chow. All experiments were performed under protocols approved by the Institutional Animal Care and Use Committee of the Feinstein Institutes for Medical Research and in strict adherence with NIH guidelines on the care and use of laboratory animals. A total of 2040 mice were used in this study.

### Vagus nerve isolation and electrical stimulation

Standard chow was withheld from animals for a period of up to 3 h prior to stimulation of the cervical vagus nerve. All surgical procedures were conducted using aseptic technique. Vagus nerve isolation was carried out as previously described (Silverman et al., 2018). Briefly, mice were administered isoflurane anesthesia through a nose cone in the supine position (oxygen flow 1 L/min, isoflurane 1.75%). An appropriate depth of anesthesia was assessed by toe pinch reflex. The cervical region was shaved with an electric razor and then sterilized with 70% ethanol. A midline incision was made, the salivary glands were identified and bluntly dissected to expose the left carotid bundle lateral to the sternocleidomastoid muscle. The left cervical vagus nerve was separated from the carotid sheath and placed on a 200 μm diameter micro cuff sling bipolar electrode with platinum-iridium contacts (CorTec GmbH, Freiburg, Germany). Parafilm was placed over the surgical site to prevent desiccation of the nerve during stimulation. Heart rate was continuously monitored using a MouseSTAT® Heart Rate Monitor (Kent Scientific, Torrington, CT, USA).

Electrical pulses were delivered by a constant current stimulator system PlexStim 2.0 (Plexon, Dallas, TX) and individually controlled with PlexStim v2.2 software. The following stimulation parameters were used for stimulation: Four minute duration, pulse width (50 μs, 250 μs), amplitude (50 μA, 200 μA, and 750 μA), and frequency (30 Hz, 100 Hz). Sham operated mice underwent the same surgical procedures but without electrical stimulation. After stimulation, the skin was sutured closed and the animals were returned to their home cages for recovery.

### Serum collection and analysis

Two hours following stimulation, whole blood was collected from animals by cardiac puncture following euthanasia by CO_2_ asphyxiation. Blood was allowed to clot in a polypropylene tube at room temperature for 30–60 min. To obtain serum, the tubes were centrifuged two times, first at 5000 x g for 10 min, followed by 10,000 x g for 2 min. The supernatant serum was collected in a clean tube and stored at -20 °C until further processing. Serum was analyzed on multiplex cytokine immunoassay plates (V-PLEX Panel 1 mouse kit; Meso Scale Discovery, Rockville MD) to quantify levels of IFN-γ, IL-1β, IL-2, IL-4, IL-5, IL-6, CXCL1, IL-10, IL-12p70, and TNF-α.

### Statistical analysis

Differences in serum cytokine levels between stimulated and non-stimulated (sham) groups were analyzed by Mann-Whitney U tests (Prism 8.0). In all tests, *P* < 0.05 was accepted as an indication of statistical significance.

## Results

**Fig. 1:**
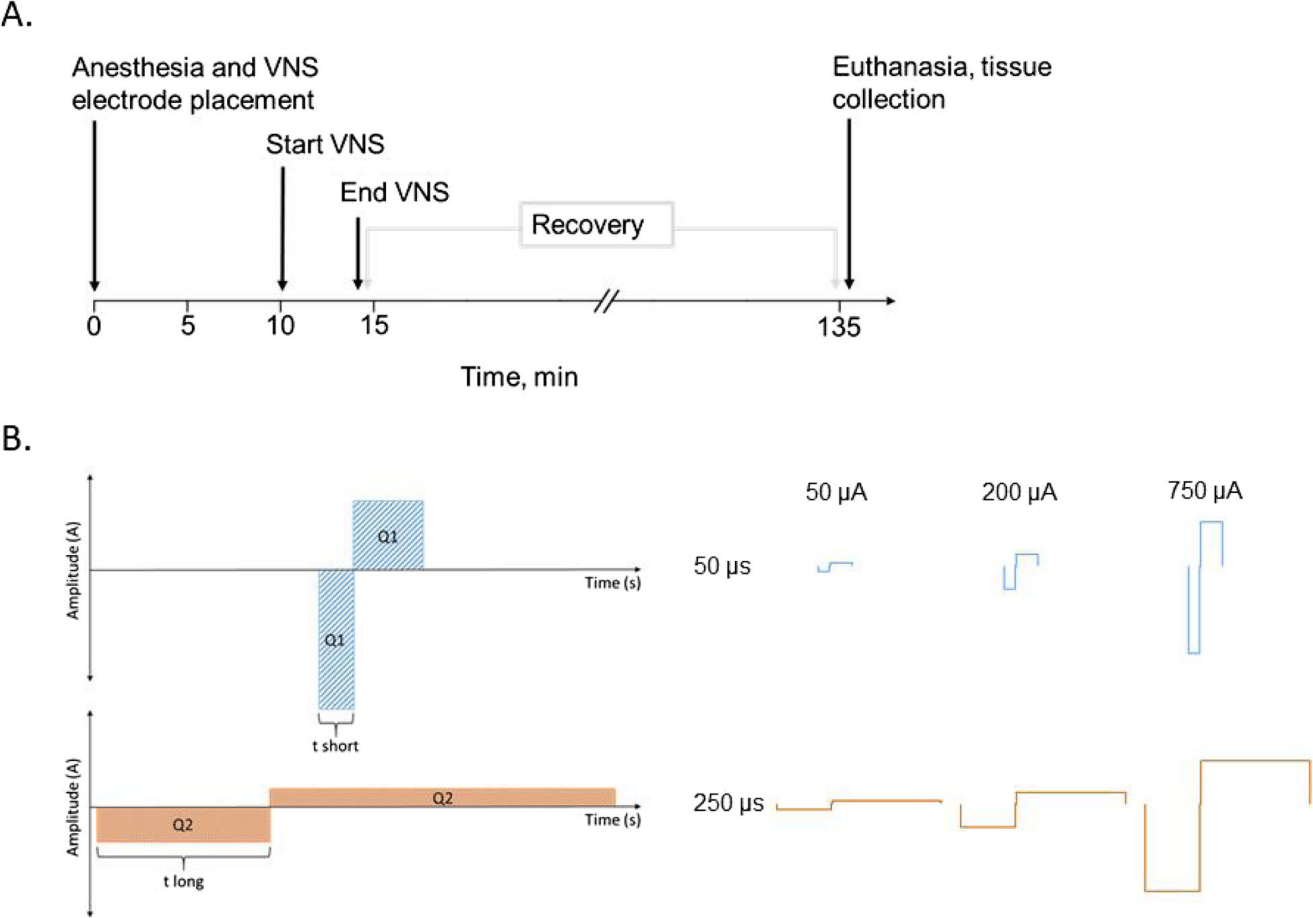
Experimental design and stimulation pulse waveforms. **a** Experimental timeline. Electrical stimulation pulse trains were applied to the exposed left cervical vagus nerve for 4 min under anesthesia. Following stimulation, the animals were recovered for 2 h, euthanized, and whole blood was collected through cardiac puncture. **b** Schematic of the charge-balanced stimulation waveforms used during stimulation, with short pulse width (top) and long pulse width (bottom). The actual waveform shapes used in this study are shown to the right

Here we utilized an established methodology to surgically isolate and electrically stimulate the vagus nerve. For all experiments, stimulation was performed on experimental animals with sham-matched controls. Following vagus nerve stimulation, mice were euthanized after 2 h, and blood was collected (Fig. 1a). During electrical stimulation, depolarization of the nerve was achieved via cathodal current application at the electrode-tissue interface and at the electrode with the lower electrical potential (1 mm spacing between electrodes). We utilized biphasic waveforms, with a secondary anodic phase to balance out the net effect charge to zero (Fig. 1b). Without charge balancing, a detrimental electrical potential buildup at the electrode may occur over time resulting in both tissue damage and reduction of electrode effectiveness. We consistently placed the electrode at the same rostral-caudal position on the vagus nerve, however, we did not monitor the cathode position on the nerve. The stimulation waveforms for the different parameters are shown in Fig. 1b.

**Fig. 2:**
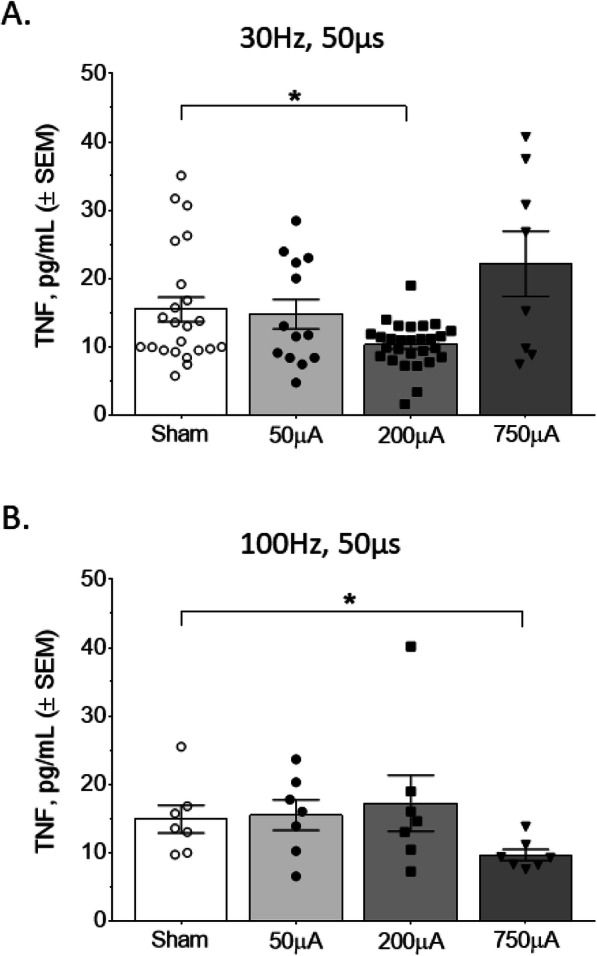
Specific stimulation amplitude and frequency combinations at 50 μs pulse widths reduce serum TNF levels. **a** A significant decrease in TNF, compared to the sham group, was observed with 30 Hz stimulation and a pulse amplitude of 200 μA. **b** A significant decrease in TNF was observed at 100 Hz stimulation with a pulse amplitude of 750 μA. Data is represented as individual mouse data points with mean ± SEM. *n* = 7–29 per group, *, *P* < 0.05

**Fig. 3:**
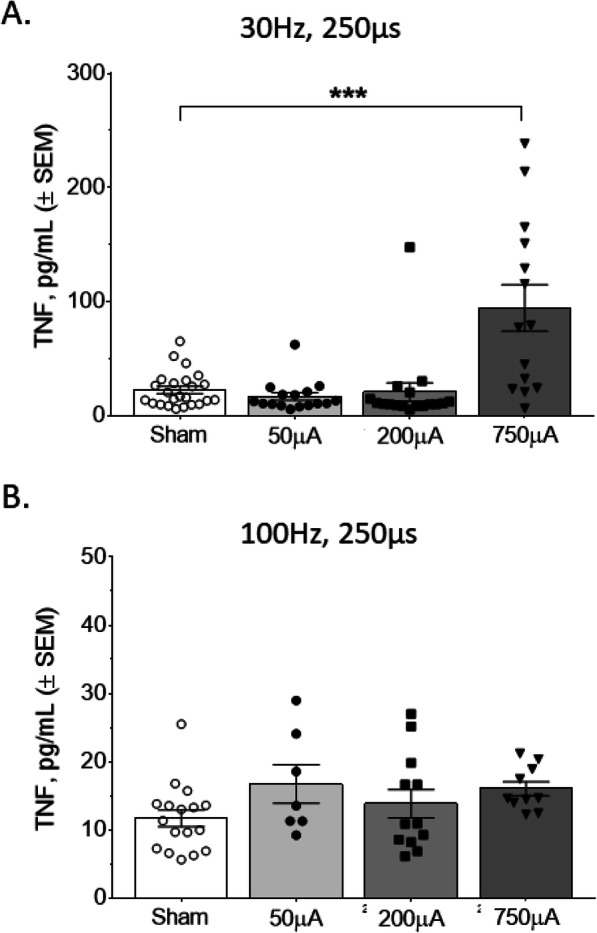
Serum TNF is significantly increased by vagus nerve stimulation at 250 μs for a specific parameter combination. **a** Stimulation resulted in a significant increase in TNF at 30 Hz and 750 μA pulse amplitude, compared to the sham group. **b** No significant changes in serum TNF were observed with the 250 μs pulse width at 100 Hz. Data is represented as individual mouse data points with mean ± SEM. *n* = 7–24 per group, *** *P* < 0.001

While electrical vagus nerve stimulation is known to decrease serum TNF in a setting of increased inflammation, its effect during normal physiology is unclear. To assess this, we delivered cervical vagus nerve stimulation to normal mice and measured serum cytokines 2 h later. We found that specific combinations of stimulation parameters significantly changed levels of serum TNF. Specifically, stimulation at the short pulse width (50 μs) at 30 Hz pulse and 200 μA amplitude produced a significant decrease in TNF (Mann Whitney U = 216, *P* < 0.05; Fig. 2a). Stimulation with a 50 μs pulse width at 100 Hz and 750 μA also resulted in a decrease in serum TNFα (Mann Whitney U = 6, *P* < 0.05; Fig. 2b). When we increased the pulse width to 250 μs during stimulation, we observed a significant increase in serum TNF levels at 30 Hz and 750 μA, compared to sham mice (Mann Whitney test, U = 60, *P* < 0.0001; Fig. 3a). In contrast, stimulation 250 μs and 100 Hz produced no statistically significant changes in serum TNF (Mann Whitney U = 38, *P* = 0.17; Fig. 3b). These results suggest that specific stimulation parameters can alter serum TNF in a bidirectional manner.

**Fig. 4:**
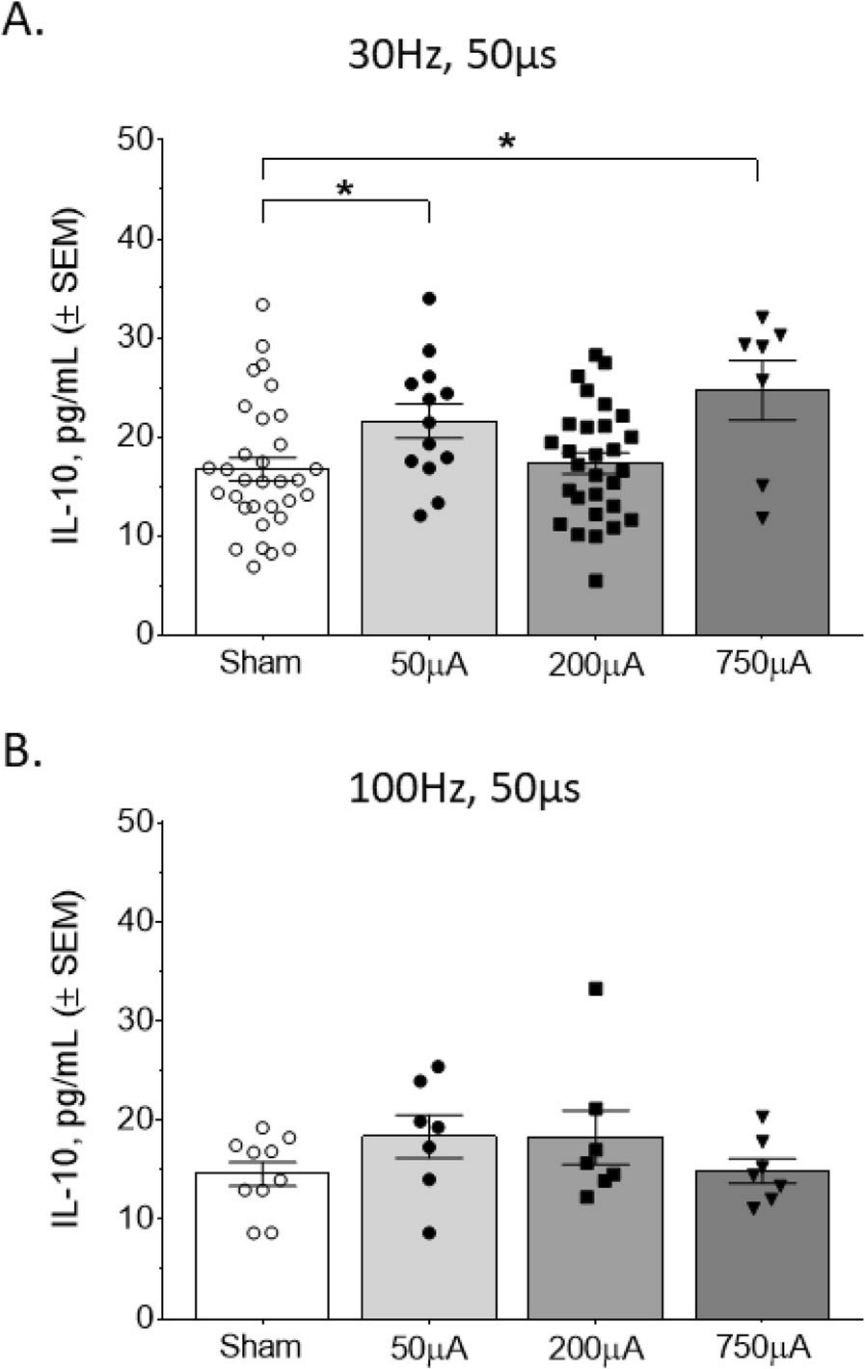
Serum IL-10 is increased by select parameters of electrical stimulation with 50 μs pulse width. **a** Stimulation for 50 μs at 30 Hz produced significant increases, compared to the sham group, in IL-10 for both 50 μA and 750 μA pulse amplitudes. **b** No changes in serum IL-10 were observed for 100 Hz stimulation across the four pulse amplitudes. Data is represented as individual mouse data points with mean ± SEM. *n* = 7–33 per group, *, *P* < 0.05

**Fig. 5:**
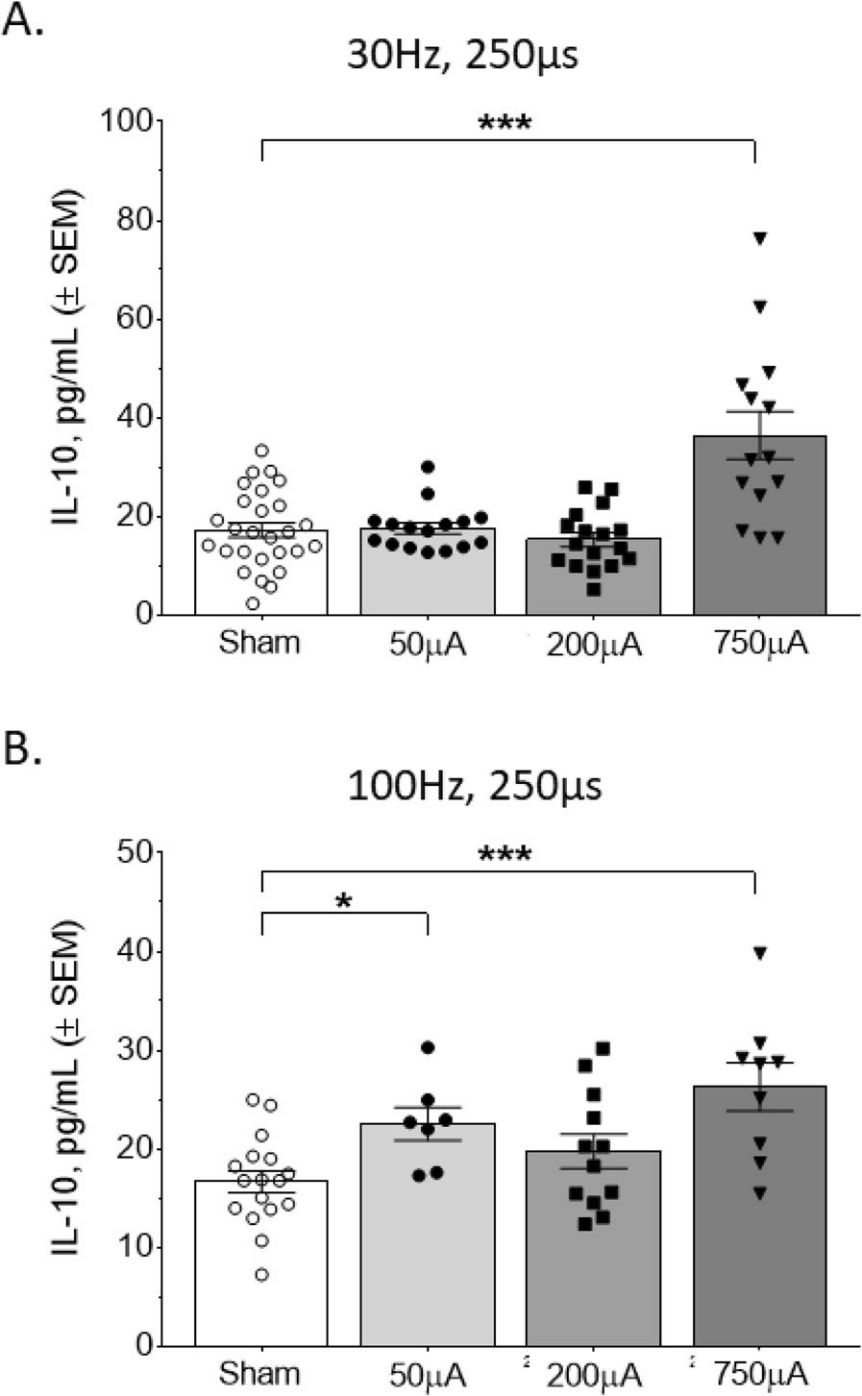
Nerve stimulation with 250 μs pulse width increased serum IL-10 at several different parameters. **a** Stimulation with 250 μs pulses at 30 Hz produced a marked increase in IL-10 at the 750 μA pulse amplitude. **b** Stimulation with 250 μs pulses at 100 Hz produced significant increases at both 50 μA and 750 μA pulse amplitudes. Data is represented as individual mouse data points with mean ± SEM. *n* = 7–27 per group, *, *P* < 0.05; *** *P* < 0.001

IL-10 is an anti-inflammatory cytokine that suppresses Th1 cells, NK cells, and macrophages during infection (Couper et al., 2008; Hutchins et al., 2013). To examine whether electrical vagus nerve stimulation could also change levels of serum IL-10, we used specific combinations of frequency, pulse width, and amplitude followed by serum IL-10 measurements. Interestingly, the shorter 50 μs pulse width at 30 Hz produced statistically significant increases in serum IL-10 at both the 50 μA and 750 μA amplitudes (50 μA, Mann Whitney U = 115, *P* < 0.05; 750 μA, Mann Whitney U = 53, *P* < 0.05; Fig. 4a). Meanwhile, a 50 μs pulse width at 100 Hz stimulation resulted in no significant changes in IL-10 levels (Mann Whitney U = 34, *P* = 0.96; Fig. 4b). With the longer 250 μs pulse width, we observed statistically significant increases in IL-10 at 750 μA amplitude for both the 30 Hz (Mann Whitney U = 53, *P* < 0.05; Fig. 5a) and 100 Hz frequencies (Mann Whitney U = 18, *P* < 0.001; Fig. 5b). There was also a significant increase in IL-10 at 100 Hz with the 250 μs pulse width at 50 μA amplitude (Mann Whitney U = 20, *P* < 0.05; Fig. 5b). This demonstrates that specific parameters have a different effect on serum IL-10, compared to TNFα.

**Fig. 6:**
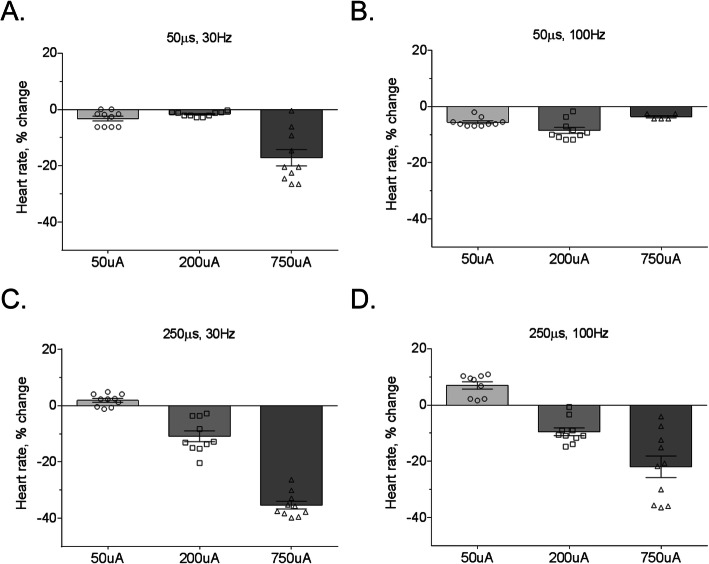
The effect of different vagus nerve stimulation parameters on heart rate. **a** Stimulation at 30 Hz with 50 μs pulse width resulted in bradycardia (≥10% reduction in heart rate) at only the 750 μA pulse amplitude. **b** At 100 Hz, bradycardia was not observed with the 50 μs pulse width. **c** Stimulation with the longer 250 μs pulse width resulted in bradycardia at 200 and 750 μA pulse amplitudes. **d** The largest decrease in heart rate was observed with the 250 μs pulse width at 100 Hz and 750 μA amplitude. Data is represented as individual mouse data points with mean ± SEM. *n* = 5–10 per group

Electrical nerve stimulation-induced bradycardia has been used to index nerve fiber activation during stimulation, specifically activation of the intermediate diameter B-fibers within the vagus nerve (Musselman et al., 2019; Yoo et al., 2013; Yoo et al., 2016). During our stimulation experiments, we measured heart rate with a continuous heart rate monitor to obtain an indirect measure of B-fiber recruitment. Stimulation with 50 μs pulses resulted in bradycardia only at the 750 μA and 30 Hz parameter (− 17.1 ± 2.9% decrease; Fig. 6a). The 50 μs pulse width stimulation did not produce bradycardia at 100 Hz for any of the tested pulse widths (Fig. 6b). With the longer 250 μs pulse at 30 Hz, bradycardia was produced at both 200 μA and 750 μA pulse amplitudes (Fig. 6c). At 100 Hz, stimulation with 250 μs pulse width produced the largest measured decrease in heart rate (− 20.4 ± 4.4%; Fig. 6d). These results indicate that alteration of stimulation parameters results in differential effects on heart rate, with longer pulse width increasing the likelihood of recruiting cardiac innervating B-fibers in the mouse vagus nerve.

In addition to assessing changes in the prototypical pro-inflammatory cytokine TNF and anti-inflammatory cytokine IL-10, we also measured serum levels of additional cytokines including: interferon gamma (IFN-γ), interleukin-12 p70 (IL-12 p70), interleukin-1 beta (IL-1β), interleukin-2 (IL-2), interleukin-4, (IL-4), interleukin-5 (IL-5), interleukin-6 (IL-6), and chemokine C-X-C motif ligand 1 (CXCL1). Serum levels of pro-inflammatory cytokines IL-6 were increased across a wide range of stimulation parameters for both the short 50 μs and long 250 μs pulse widths (Table 1, Table 2). Serum CXCL1 was also significantly increased by stimulation across several parameter combinations. For anti-inflammatory cytokines, we did not observe changes in IL-4 for any stimulation parameter combination. These results indicate that electrical stimulation may exert differential effects on the modulation of cytokines. It should be noted that the measured concentrations of IFNγ, IL-1β, IL-2, IL-4, and IL-5 were very low and close to the sensitivity limits for the ELISA kit. Although there may be statistically significant changes with some of the stimulation parameters for these cytokines, these low levels may lack physiological relevance (Table 1, Table 2).

**Table 1:**
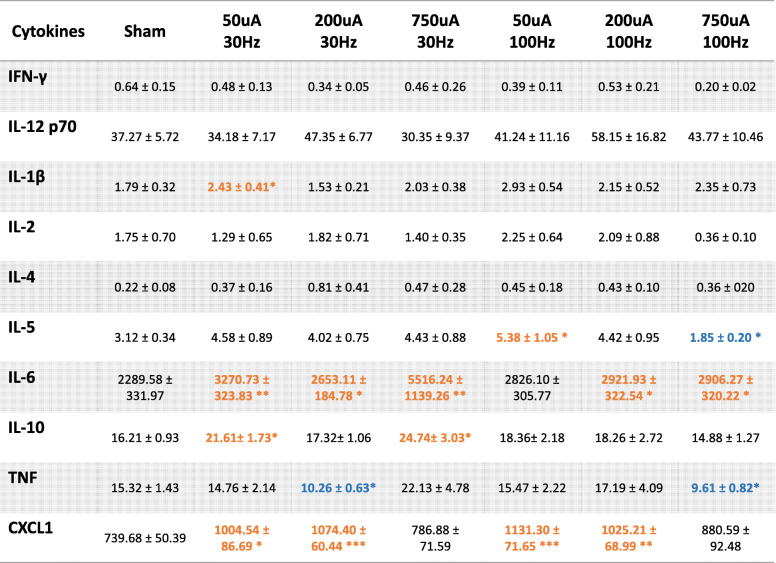
Cytokines (mean ± SEM) at 50 μs pulse

Full multiplex panel results of serum cytokine levels following nerve stimulation with 50 μs pulse width. Values in orange indicate significant increases while values in blue indicate significant decreases, compared to the Sham group. *n* = 7–29 per group; *, *P* < 0.05; **, *P* < 0.01; *** *P* < 0.001

**Table 2:**
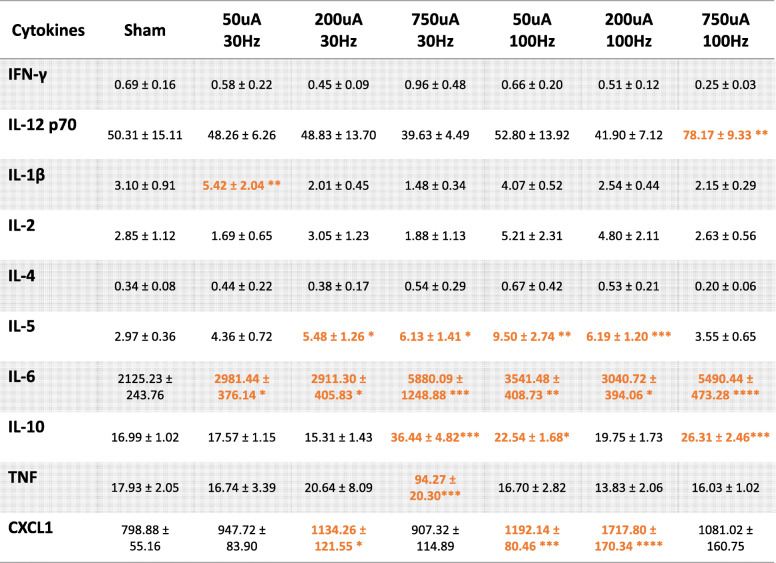
Cytokines (mean ± SEM) at 250 μs pulse

Full multiplex panel results of serum cytokine levels following nerve stimulation with 250 μs pulse width. Values in orange indicate significant increases, compared to the Sham group. *n* = 9–33 per group; *, *P* < 0.05; **, *P* < 0.01; *** *P* < 0.001; ****, *P* < 0.0001

## Discussion

The neural control of immunity through stimulation of the vagus nerve holds significant promise for treating inflammatory disorders. Here we have shown that a set of electrical stimulation parameters can change specific cytokine levels in the absence of inflammation. To our surprise, we found electrically stimulating the vagus nerve with specific parameters results in increased circulating levels of TNF and IL-10, cytokines known to be involved in inflammatory responses. These results indicate that systemic cytokines respond to specific combinations of frequency, amplitude, and pulse width applied to the cervical vagus nerve. How these parameters affect the recruitment of specific fiber types within the vagus nerve remains a topic of important ongoing work. Our results indicate that the refinement of these parameters may be important in the neuromodulation of immunological responses mediated through vagal signaling.

The effect of vagus nerve stimulation on cytokines in the absence of systemic inflammation has not, to our knowledge, been carefully examined. This is the first demonstration that specific stimulation parameters can be used to increase serum TNF and IL-10 levels. TNF is an inflammatory cytokine that is released by macrophages following infection and injury. Transient elevations in serum TNF are required to coordinate host defense against pathogens and the repair of injured host tissue. While physiological inflammation is locally protective, pathological inflammation caused by persistent and unregulated TNF levels cause chronic tissue damage and drive the pathogenesis of inflammatory disease including rheumatoid arthritis, Crohn’s disease, and inflammatory bowel disease (Bonaz et al., 2016; Kalliolias 2016; Koopman et al., 2016). A large body of work established the critical role of TNF in the pathogenesis of systemic inflammation in animal models and led to the clinical translation of TNF blockade to treat chronic inflammation. The beneficial effect of vagus nerve stimulation for reducing TNF in the context of inflammatory disorders is clear, however, an increase in proinflammatory cytokine levels may also be beneficial in certain physiological and pathologic contexts. An increase in circulating cytokines might be useful to bolster the immune system in conditions of immunosuppression, for example, due to immunodeficiencies or viral infection (Breen, 2002; Varzaneh et al., 2014). Cancer patients with lower levels of circulating cytokines may also benefit from selective neuromodulation techniques to increase those levels (Khan et al., 2019). As techniques are developed to better understand the relationship between vagus nerve activity and cytokine signaling (Steinberg et al., 2016; Tsaava et al., 2019; Zanos et al., 2018), there may also be other physiological conditions that would benefit from intentionally increasing cytokine levels.

Our results suggest that serum cytokines may likewise be controlled by specific fiber sets and firing patterns. Altering the electrical stimulation parameters affects the recruitment of specific vagus nerve fibers. A large body of evidence shows that increasing pulse width and amplitude, and consequently the charge delivered during both cathodic and anodic phases of the pulse, inhibits activation of large diameter fibers while selectively activating smaller diameter fibers (Baratta et al., 1989; Musselman et al., 2019). For example, lower amplitude stimulation activates A-fibers but as stimulation increases, the A-fiber activation becomes suppressed and B-fibers become activated to change heart rate (Burke et al., 1975). It is possible that the differential effect on heart rate may be a result of effective recruitment of large and small fiber types with the long pulse at lower amplitudes, and selective inhibition of larger fibers at the larger pulse widths. The effective pulse widths and stimulus amplitudes necessary to elicit heart rate changes is shown in Fig. 6. As the stimulation charge increased, we generally observed more pronounced bradycardia, however, heart rate decreases were not always coupled to changes in serum cytokines. The largest increase in serum TNF at 250 μs pulse width, 30 Hz, and 750 μA was associated with the largest decrease in heart rate. However, TNF levels did not change for two other stimulation parameters that induced bradycardia (Fig. 6: 50 μs pulse width, 30 Hz, 750 μA and 250 μs pulse width, 100 Hz, 750 μA). This dissociation between bradycardia and serum cytokines levels confirms prior work indicating that cytokine effects are independent of the B-fiber activation that induces bradycardia (Huston et al., 2007). While the specific vagus nerve fibers mediating the inflammatory reflex are not known, they are thought to be cholinergic efferents descending to peripheral ganglia (Bonaz et al., 2013; Chavan et al., 2017). These cholinergic fibers are myelinated and intermediate in diameter (i.e. A- or B-fibers), and are therefore targeted for recruitment by stimulation pulses of moderate width and amplitude (Olofsson et al., 2015).

We have considered the possibility that different durations of total stimulation may also be an important variable in cytokine-specific modulation via the vagus nerve. The frequency of stimulation also plays a role in fiber recruitment. While not examined in this study, lower frequency stimulation (1–20 Hz) may also alter cytokine levels. Further, while the applicability of these findings may be most relevant for disease conditions, these studies were carried out in healthy control animals. Therefore, they should be interpreted with caution if trying to extrapolate to specific disease conditions. The parameter space of electrical nerve stimulation is important because varying time, duration, and amplitude should affect activation of different target organs and brain structures. Electrical stimulation delivered to cervical vagus nerve is broadly applied to all nerve fibers, including sensory afferents and motor efferents. It is thought that B-fibers mediate motor efferent control of the visceral organs. Because the vagus nerve innervates several major organs in the viscera, stimulation of this neve has the potential to treat a broad range of disorders ranging including but not limited to arthritis, myocardial infarctions, and metabolic disorders (Kong et al., 2012; Levine et al., 2014; Chang et al., 2019).

## Conclusions

The unexpected results here indicate that it is possible to stimulate the production of TNF and other cytokines using electrical stimulation of the cervical vagus nerve in normal, non-inflammatory conditions. As bioelectronic therapies continue to evolve for controlling inflammation, increasingly refined stimulation strategies will be needed to activate fibers of interest (Grill, 2015; Tsaava et al., 2019), while also minimizing the potential for the negative outcomes and off-target effects associated with vagus nerve stimulation. The effectiveness of these selective stimulation paradigms will depend on knowing which specific fiber populations mediate the neural regulation of inflammation and the optimal techniques for activating them. Our results show that varying stimulation parameters of frequency, amplitude, and pulse width holds promise for the specific regulation of cytokine responses in both health and disease.

## Data Availability

Datasets used in this study are available from the corresponding author upon reasonable request.

## Abbreviations

CXCL1: Chemokine ligand C-X-C motif ligand 1
IFNγ: Interferon gamma
IL10: Interleukin-10
IL-12 p70: Interleukin-12 p70
IL-1β: Interleukin-1 beta
IL-2: Interleukin-2
IL-4: Interleukin-4
IL-5: Interleukin-5
IL-6: Interleukin-6
TNFα: Tumor necrosis factor alpha
